# Exercise, Dietary Habits, and Defecatory Dysfunction in Patients Living with Colorectal Cancer: A Preliminary Quantitative Study

**DOI:** 10.3390/healthcare12111136

**Published:** 2024-06-03

**Authors:** Hiromi Nakagawa, Hiroyuki Sasai, Yoshimi Kato, Shinobu Matsumoto, Kiyoji Tanaka

**Affiliations:** 1 Graduate School of Medicine, Gifu University, Gifu 501-1194, Japan; 2 Research Team for Promoting Independence and Mental Health, Tokyo Metropolitan Institute for Geriatrics and Gerontology, Tokyo 173-0015, Japan; sasai@tmig.or.jp; 3 Uji-Tokushukai Medical Center, Kyoto 611-0041, Japan; 4 Medical Research Institute Kitano Hospital, Osaka 530-8480, Japan; 5 Faculty of Health and Sport Sciences, University of Tsukuba, Tsukuba 305-8577, Japan; tanaka.kiyoji.ft@u.tsukuba.ac.jp

**Keywords:** colorectal cancer, survivors, defecatory dysfunction, exercise habits, diet

## Abstract

This study investigated the association of exercise and dietary habits with defecatory dysfunction in patients living with colorectal cancer. We recruited 61 adult patients who had undergone surgery within the past 20 years and attended outpatient clinics at designated cancer hospitals in Japan. Defecatory dysfunction was defined as any symptom caused by issues with colon and anal function, including fecal incontinence, evacuation difficulties, frequent stools, diarrhea, and constipation. Exercise and dietary habits were assessed via a quantitative questionnaire survey. Postoperative defecatory dysfunction occurred in all the patients. Multivariate analysis revealed no association between exercise habits and defecatory dysfunction; however, dietary fiber intake ≥4 times a week was associated with frequent stools (adjusted odds ratio, 5.11; 95% confidence interval, 1.10, 23.70). These findings suggest a need to alleviate defecatory dysfunction by improving one’s dietary habits. Interventions aimed at alleviating defecatory dysfunction by improving the dietary habits in patients living with colorectal cancer are needed.

## 1. Introduction

Colorectal cancer (CRC) is the second most common cancer worldwide, with an estimated 3.2 million cases projected by 2040 [1,2]. Maintaining the activities of daily living and improving the quality of life of patients living with CRC [3] are ongoing challenges, particularly because defecatory dysfunction often arises after anus-preserving surgery [4,5]. Furthermore, low anterior resection syndrome occurs in approximately 80–90% of the cases after anus-preserving surgery for rectal cancer and can even persist in patients for more than 10 years post-surgery [6]. Among these complications, defecatory dysfunction has emerged as a frequently reported but inadequately addressed issue. The efforts to ameliorate such postoperative issues have seen potential advancements in exercises targeting pelvic floor muscle strength and dietary modifications [7,8,9,10,11,12,13]. Physical activity and exercise can also enhance insulin sensitivity, improve anti-cancer immune function, promote gastrointestinal motility, and reduce the exposure to carcinogenic substances in the digestive tract. Exposure to carcinogens indicates the continued accumulation of secondary bile acids in the intestinal tract, which is an important risk factor for gastrointestinal cancer [14]. Therefore, shortening the transit time of carcinogenic substances in the digestive tract reduces the risk of colorectal cancer [15]. 

The recommendations from the 2018 World Cancer Research Fund International/National Cancer Institute report [16] and American Society of Clinical Oncology (ASCO) guidelines [17] emphasize the importance of cancer survivors increasing their engagement in moderate to vigorous physical activity and reducing sedentary behavior. Moreover, engaging in aerobic exercise and strength training for approximately 30 min per session, at least five times a week, for a total of 150 min of exercise, has been shown to alleviate the common side effects of cancer treatment and improve health.

Several lifestyle factors contribute to the development of CRC, including a high intake of general and processed red meat and a low intake of fruits and vegetables. Dietary fiber intake may counter some of the carcinogenic effects of bile acids [15]. Dietary modifications are considered a strategy to adjust defecatory dysfunction [18]. Furthermore, defecation volume significantly decreases with a fluid intake of 500 mL or less per day [19]. Some studies have investigated the dietary modifications and defecatory conditions in patients with CRC. However, there is a dearth of studies that have examined the association between meal frequency, red meat and fiber intake, fluid intake, and defecatory dysfunction [20,21,22,23].

The American Cancer Society (ACS) and World Cancer Research Fund/American Institute for Cancer Research (WCRF/AICR) recommend adopting a physically active lifestyle and consuming a healthy diet with an emphasis on plant-based foods [24]. The adherence to cancer prevention guidelines for diet and physical activity has been associated with a significant decrease in the incidence of CRC [24]; however, research examining the relationship between lifestyle habits (exercise and diet) and defecatory dysfunction in patients with CRC remains limited.

The research question for this study was, “Are exercise and diet associated with defecatory dysfunction in patients with a history of CRC?”. With this, we aimed to investigate the association between lifestyle habits and postoperative defecatory dysfunction in patients with CRC using a preliminary quantitative questionnaire-based survey. Overall, this preliminary study provides foundational data for the development of a combined exercise and dietary intervention program aimed at alleviating the defecatory dysfunction in patients living with CRC.

## 2. Materials and Methods

### 2.1. Study Design, Setting, and Patients

This multicenter, cross-sectional study employed a quantitative preliminary survey using an anonymous self-administered questionnaire. The questionnaire survey was conducted from October 2022 to March 2023. The questionnaire was developed based on the ACS recommendations and adherence score [24], the ASCO guidelines [17], and our previous qualitative study [25]. Patients with cancer stages I to IV based on the tumor, node, and metastasis classification system and a history of CRC were randomly recruited from the outpatient units at two designated cancer hospitals in Japan. The physician and certified wound, ostomy, and continence nurse (WOCN) received written informed consent from all patients. The inclusion criteria were patients who had undergone CRC surgery within the past 20 years and whose surgical records were available. Patients were defined as those who had no locoregional CRC recurrence and were judged by the physician and WOCN as requiring outpatient care. The exclusion criteria included patients undergoing radiation therapy and chemotherapy at the time of study entry, those with psychiatric dysfunction, severe arrhythmias, or undergoing dialysis, and those with a history of or who were currently undergoing dementia treatment. The patients’ exercise, diet, and defecatory dysfunction status at the time of the survey were investigated.

### 2.2. Measurement Parameters

#### 2.2.1. Primary Outcomes

Defecatory dysfunction was defined as symptoms primarily caused by colorectal issues, including fecal incontinence (FI) [4,5,26], evacuation difficulties (ED), frequent stools (FS), diarrhea, and constipation. The definitions and assessment criteria used to evaluate defecatory dysfunction are shown in Table 1. 

For FI, the investigation focused on anal FI and excluded incontinence or leakage from the stoma. In addition, we investigated whether the patients wore diapers or incontinence pads because of the involuntary leakage of feces from the anus. Constipation was classified based on the Bristol Stool Form (BS) scale scores. The BS scale is an index that classifies bowel movements into seven categories based on stool shape and hardness. It is used as a diagnostic criterion for constipation and diarrhea. Generally, BS scale scores of 1 and 2 indicate constipation, 3–5 indicate healthy stool types, and 6 and 7 indicate diarrhea [26]. The presence and frequency of defecatory dysfunction, stool characteristics, and BS scale scores were investigated using medical records and questionnaires. Multiple responses regarding the symptoms of defecatory dysfunction were allowed in the questionnaire.

#### 2.2.2. Exposure Variables

The exposure variables were exercise and dietary habits. We assessed exercise habits, which were defined as exercising for more than 150 min per week. We asked the participants about the exercise duration per session and the number of times that they exercised per week, and the weekly exercise duration was calculated accordingly. Additionally, we inquired about the exercise types that they enjoyed. We also assessed the number of daily meals and their contents (frequency of red meat intake, dietary fiber intake per week, and foods that tend to cause diarrhea or constipation problems) and daily fluid intake. Red meat was defined as any red meat, regardless of whether it was processed or unprocessed. Dietary fiber was defined as fruits, vegetables, and grains [24]. The number of meals per day was categorized as two or more and less than two meals per day, and daily fluid intake was categorized as 1 L or more or less than 1 L [19]. The data on exercise and diet were collected from medical records and questionnaires.

#### 2.2.3. Other Patient Characteristics

We collected the data on patient characteristics, including age, sex, weight, postoperative weight gain or loss, body mass index, medical history, employment, and alcohol and smoking history. Hemoglobin A1c values were collected to represent dietary habits. Information on the cancer stage, date of surgery, surgical procedures, surgical site, and medication were collected as variables potentially influencing defecatory dysfunction. The data on these patient characteristics were obtained from medical records.

### 2.3. Statistical Analyses

The data were analyzed using descriptive and Chi-squared tests. Additionally, multiple logistic regression analysis was performed with confounding adjustment for age [32], sex, and the site of surgery, which were considered to influence defecatory dysfunction. Although there are many other confounding factors, we prioritized these three established confounders, considering the limited sample size. Adjusted odds ratios (AORs) and 95% confidence intervals (CIs) were utilized to assess the associations between lifestyle habits and defecatory dysfunction. Statistical analysis was performed using the SPSS Statistics version 27 for Windows software (IBM Corp., Chicago, IL, USA), with statistical significance set at *p* < 0.05.

## 3. Results

### 3.1. Patient Characteristics

**Figure 1:**
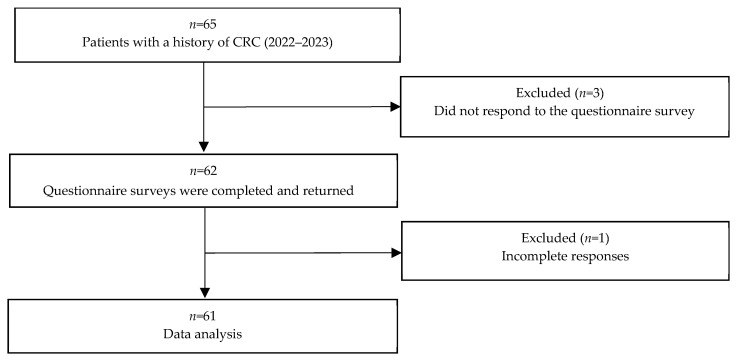
Flowchart of the study patients. CRC, colorectal cancer.

Of the 65 patients living with CRC who gave their consent and received the questionnaire survey, 61 provided complete data (valid response rate of 93.8%). The patient flow chart is shown in Figure 1. Table 2 shows the patients’ demographic characteristics.

### 3.2. Exercise Habits and Defecatory Dysfunction

In this study, 22 (36.0%) patients met the recommended exercise duration of 150 min per week. The median (minimum, maximum) exercise duration per week was 90 (0, 630) min. Walking was the most common type of exercise (*n* = 34, 87.2%), followed by stationary cycling (*n* = 2, 5.1%), golf (*n* = 2, 5.1%), strength training (*n* = 2, 5.1%), and ballroom dancing (*n* = 1, 2.6%). 

Postoperative defecatory dysfunction occurred in all patients. Bulk-forming laxatives were used in patients whose rectum was the surgical site. Patients experienced two to four symptoms of defecatory dysfunction. The data related to postoperative exercise habits and defecatory dysfunction are shown in Table 3. There was no significant correlation between postoperative exercise habits and defecatory dysfunction. Postoperative defecatory dysfunction manifested as FI in 25 patients (41.0%), ED in 24 patients (39.3%), FS in 18 patients (29.5%), diarrhea in 17 patients (27.9%), and constipation in 9 patients (14.7%). 

### 3.3. Dietary Habits and Defecatory Dysfunction 

Twelve patients (19.7%) consumed <2 meals per day (Table 4). Among the patients, 15 (24.6%) reported consuming red meat ≥4 times per week (Table 4), and 38 (62.3%) reported consuming dietary fiber ≥4 times per week (Table 5). None of the patients simultaneously consumed both diarrhea- and constipation-inducing dietary fiber. However, milk, fatty foods, and garlic were identified as foods that triggered diarrheal symptoms. None of the patients consumed probiotics as part of their diet. Table 4 and Table 5 present the data related to postoperative dietary habits and defecatory dysfunction. Forty patients (65.6%) consumed <1 L of fluid per day. 

Patients who consumed red meat ≥4 times per week had more frequent stools than those who consumed red meat <4 times per week (*χ*^2^ = 8.89, *p* < 0.01). Similarly, patients with a fluid intake of <1 L per day had more frequent stools than those with a fluid intake of ≥1 L per day (*χ*^2^ = 6.15, *p* < 0.05). The patients with constipation reported a daily fluid intake of <1 L (*χ*^2^ = 5.29, *p* < 0.05). Patients who ate fewer than two meals per day had a higher incidence of diarrhea than those who ate more than two meals per day (*χ*^2^ = 6.90, *p* < 0.05). 

### 3.4. Exercise, Dietary Habits, and Defecatory Dysfunction

Multiple logistic regression analysis was used to assess the associations between exercise habits, meal frequency, red meat intake, dietary fiber intake, and fluid intake after adjusting for sex, age, and the surgical site [33,34] (Table 6). We did not use multiple logistic regression analysis to evaluate constipation because of the limited number of patients with the symptom and insufficient statistical power.

The patients who consumed dietary fiber ≥4 times per week had significantly higher odds for FI (*p* = 0.04, AOR, 5.11; 95% CI, 1.10, 23.70). 

## 4. Discussion

This study investigated the relationship between exercise habits and dietary and defecatory dysfunction in patients with CRC using a self-report questionnaire. No significant association was found between exercise habits and defecatory function; however, the patients who often consumed dietary fiber were more likely to experience frequent stools. These findings suggest a need to alleviate defecatory dysfunction by improving one’s dietary habits. 

According to an ASCO survey [17], 56.8% of the responders engaged in physical activity [35]. However, only 36% of the patients in the present study engaged in exercise at the recommended level. Based on the results of this study, defecatory dysfunction was not associated with exercise habits. 

In our unadjusted models, the patients who consumed red meat ≥4 times a week and had a fluid intake of <1 L per day were more likely to experience FS. The patients with a meal frequency of <2 times/day experienced diarrhea and may have had inadequate fluid intake. Greenberg et al. [36] reported that a lower red meat intake and higher fruit and vegetable intake were associated with lower FS (better bowel function). We observed more FS in the patients who consumed fiber four or more times per week. Soluble dietary fiber softens stools and regulates stool consistency by dissolving them in and forming a gel with fluid. Moreover, insoluble dietary fiber absorbs fluid, activates intestinal peristalsis, and improves bowel movements [37]. Adequate fluid intake is also important when consuming fiber-rich foods. Without adequate fluid intake, fibers cannot expand in the intestine and may cause constipation. Therefore, it is important to consume fluid concomitantly with dietary fiber. Furthermore, unlike saline laxatives, which draw fluid from the body without being absorbed in the intestinal tract, bulk-forming laxatives absorb a large amount of fluid in the gastrointestinal tract, stimulating the intestine and enhancing reflexive movements. Thus, when coupled with insufficient fluid intake, bulk-forming laxative administration can worsen constipation and lead to intestinal obstruction, dehydration, and other potential complications such as ED and FS [38]. 

Additionally, to prevent CRC, processed meat consumption must be limited to <76 g per day [39]. Patients who consume red or processed meat ≥4 times per week have been reported to have a 20% higher risk of developing CRC than those who consume red or processed meat <2 times per week [40]. Exercise and dietary interventions to mitigate constipation and stimulate gastrointestinal motility are recommended to reduce the exposure to carcinogenic substances in the digestive tract and lower the risk of gastrointestinal cancers through a shortened stool transit time [7,14,15]. In the present survey, it was deemed necessary to provide dietary information on the prevention of the recurrence of CRC. These findings suggest the need to alleviate the symptoms caused by defecatory dysfunction through appropriate dietary habits.

This study has some limitations. First, the outpatients were recruited randomly, leading to unavoidable sampling bias. Although our findings did not indicate a relationship between dietary habits and defecatory dysfunction, large-scale investigations applying the insights gained from the preliminary survey are necessary to ensure generalizability. Second, a questionnaire survey may have introduced recall bias and compromised accuracy. Third, the limited prevalence of constipation and insufficient statistical power did not allow us to conduct multiple logistic regression analyses with these two outcomes. We are currently addressing this issue with a larger sample size guided by preliminary study outcomes. Although we investigated exercise and defecatory dysfunction, future research should aim to eliminate bias by analyzing exercise intensity and step count data using physical activity monitors. Further investigation is needed on the number of calories consumed, the type of diet, and the type of exercise (whether aerobic or anaerobic), which were not investigated in this study. In addition, future studies should apply the insights gained from this study and further explore the relationship between exercise, dietary habits, and defecatory dysfunction to develop appropriate lifestyle intervention programs.

## 5. Conclusions

Although the association between exercise habits and defecatory dysfunction was not significant, dietary habits were associated with defecatory dysfunction. Specifically, consuming dietary fiber ≥4 times a week was associated with frequent stools. Therefore, interventions targeting dietary modification to alleviate defecatory dysfunction are necessary for patients with a history of CRC.

## Figures and Tables

**Table 1:** Definition and assessment of defecatory dysfunction.

	Definition	Assessment
Fecal incontinence	Patients with uncontrolled passage of feces or gas for at least 1 month, who had previously achieved control [4,26].	The stool leaks from the rectum without warning [26]. The patients were surveyed to determine whether they wore diapers or incontinence pads.
Evacuation difficulties	A condition where there is a frequent urge to defecate, but the act of evacuation is difficult [27,28].	The patient has inadequate rectal evacuation of feces and residual stools and senses the presence of stools and the need to defecate but is unable to do so.
Frequent stools	A condition in which a patient defecates (eliminates waste from the bowel) more often than usual [29].	The patient defecates >4 times a day.
Diarrhea	The frequent passage of loose stools with urgency (or more frequent passage than what is normal for the individual).	The passage of more than three unformed stools in 24 h [30].
Constipation	Bowel symptoms (difficult or infrequent [>3 days per week] passage of stools, hardness of stools, or a feeling of incomplete evacuation) that occur either in isolation or secondary to another underlying dysfunction [31].	The BS scale [26] was used to investigate constipation continence. The presence of constipation was investigated using medical records and questionnaires.

BS, Bristol Stool Form.

**Table 2:** Demographic characteristics of the patients.

Item		*n* = 61
Sex	M	42 (68.8%)
	F	19 (31.2%)
Age	Years	66.7 ± 12.9
Employed		18 (29.6%)
Smoking		9 (14.7%)
Alcohol		12 (19.7%)
Weight		57.4 ± 12.6
BMI	kg/m^2^	22.6 ± 4.5
	<18.5 underweight	11 (18.0%)
	>25.0 overweight/obese	12 (19.7%)
Postoperative weight gain or loss	kg	−0.6 ± 0.8
Surgical site	Rectal	50 (82.0%)
	Colon	11(18.0%)
TNM-UICC stage	I	13 (21.3%)
	II	17 (27.9%)
	III	22 (36.1%)
	IV	6 (9.8%)
	Unknown	3 (4.9%)
Operative procedure	LAR	32 (52.5%)
	Colectomy	11 (18.0%)
	ISR	1 (1.6%)
	Others	17 (27.9%)
Medical history	Diabetes mellitus	17 (27.9%)
	Hypertension	21 (34.4%)
	Stroke	5 (8.2%)
	Angina pectoris	6 (1.0%)
	Dyslipidemia	8 (13.1%)
	Respiratory illness	8 (13.1%)
	History of fractures	12 (19.7%)
Medication	Laxatives	13 (21.3%)
	Antidiarrheal	9 (14.7%)
Physiological data	HbA1c (%)	6.1 ± 1.0

Data are presented as *n* (%) or mean ± standard deviation. BMI, body mass index according to the World Health Organization classification; HbA1c, hemoglobin A1c; ISR, intersphincteric resection; LAR, low anterior resection; TNM-UICC, tumor-node-metastasis staging according to the Union for International Cancer Control. The term “laxatives” refers to bulk-forming laxatives.

**Table 3:** Prevalence of defecatory dysfunction by postoperative exercise habits.

	Exercise Habits		
	<150 min/Week (*n* = 39)	≥150 min/Week (*n* = 22)	*p*
Fecal incontinence	17 (43.6%)	8 (36.4%)	0.79
Evacuation difficulties	15 (38.5%)	9 (40.9%)	1.00
Frequent stools	11 (28.2%)	7 (31.8%)	0.78
Diarrhea	13 (33.3%)	4 (18.2%)	0.25
Constipation	5 (12.8%)	4 (19.0%)	0.71

Data are presented as *n* (%). min, minute.

**Table 4:** Prevalence of defecatory dysfunction by meal frequency and red meat intake.

	Meal Frequency			Red Meat Intake		
	<2 Times/Day (*n* = 12)	≥2 Times/Day (*n* = 49)	*p*	<4 Times/Week (*n* = 46)	≥4 Times/Week (*n* = 15)	*p*
Fecal incontinence	6 (50.0%)	19 (38.8%)	0.53	19 (41.3%)	6 (40.0%)	1.00
Evacuation difficulties	5 (41.7%)	19 (38.8%)	1.00	17 (37.0%)	7 (46.7%)	0.55
Frequent stools	5 (41.7%)	13 (26.5%)	0.31	9 (19.6%)	9 (60.0%)	<0.01
Diarrhea	7 (58.3%)	10 (20.4%)	0.03	12 (26.1%)	5 (33.3%)	0.74
Constipation	0 (0.0%)	9 (18.8%)	0.18	8 (17.4%)	1 (7.1%)	0.67

Data are presented as *n* (%).

**Table 5:** Prevalence of defecatory dysfunction by dietary fiber and fluid intake.

	Dietary Fiber Intake			Fluid Intake		
	<4 Times/Week (*n* = 23)	≥4 Times/Week (*n* = 38)	*p*	<1 L/Day (*n* = 40)	≥1 L/Day (*n* = 21)	*p*
Fecal incontinence	12 (52.2%)	13 (34.2%)	0.19	18 (45.0%)	7 (33.3%)	0.42
Evacuation difficulties	10 (43.5%)	14 (36.8%)	0.79	18 (45.0%)	6 (28.6%)	0.28
Frequent stools	4 (17.4%)	14 (36.8%)	0.15	16 (40.0%)	2 (9.5%)	0.02
Diarrhea	8 (34.8%)	9 (23.7%)	0.39	13 (32.5%)	4 (19.0%)	0.37
Constipation	3 (13.0%)	6 (16.2%)	1.00	9 (22.5%)	0 (0.0%)	0.02

Data are presented as *n* (%).

**Table 6:** Exercise habits, dietary habits, and defecatory dysfunction.

	Fecal Incontinence	Evacuation Difficulties	Frequent Stools	Diarrhea
No exercise habits <150 min/week (vs. ≥150 min/week)	1.22 (0.36, 4.18)	1.33 (0.40, 4.45)	4.43 (0.86, 22.90)	0.70 (0.15, 3.20)
Meal frequency <2 times/day (vs. ≥2 times/day)	0.90 (0.22, 3.68)	0.94 (0.23, 3.83)	0.84 (0.18, 3.84)	3.88 (0.84, 17.9)
Red meat intake ≥4 times/week (vs. <4 times/week)	1.15 (0.49, 2.69)	1.59 (0.60, 4.23)	1.20 (0.49, 2.94)	0.47 (0.09, 2.29)
Dietary fiber intake ≥4 times/week (vs. <4 times/week)	0.52 (0.17, 1.60)	0.73 (0.24, 2.20)	5.11 (1.10, 23.70)	0.54 (0.15, 1.99)
Fluid intake <1 L/day (vs. ≥1 L/day)	1.20 (0.37, 3.94)	1.86 (0.56, 6.17)	4.17 (0.77, 22.90)	1.18 (0.27, 5.10)

All models were adjusted for sex, age, and the surgical site. Adjusted odds ratios and 95% confidence intervals are shown.

## Data Availability

The study data are available upon request from the corresponding author.
